# 3-(Pyridin-2-yl)coumarin

**DOI:** 10.1107/S1600536810039796

**Published:** 2010-10-20

**Authors:** Yu-Xia Da, Zheng-Jun Quan

**Affiliations:** a Gansu Key Laboratory of Polymer Materials, College of Chemistry and Chemical Engineering, Northwest Normal University, Lanzhou 730070, People’s Republic of China

## Abstract

In the title compound, C_14_H_9_NO_2_, the dihedral angle between the pyridine ring and the lactone ring is 10.40 (3)°. The coumarin ring system is nearly planar, with a dihedral angle of 1.40 (2)° between the lactone and benzene rings. An intra­molecular C—H⋯O hydrogen bond occurs. In the crystal, inversion dimers linked by pairs of C—H⋯O inter­actions occur, generating *R*_2_^2^(14) loops.

## Related literature

For background to the structures and properties of coumarins, see: Fylaktakidou *et al.* (2004 ▶); Griffiths *et al.* (1995 ▶); Moffett (1964 ▶); Ren & Huo (2008 ▶); Ren *et al.* (2010 ▶); Trenor *et al.* (2004 ▶); Walshe *et al.* (1997 ▶); Yu *et al.* (2006 ▶); Yu, Yang *et al.* (2010 ▶); Yu, Zhang *et al.* (2010 ▶). For reference bond lengths, see: Allen *et al.* (1987 ▶). 
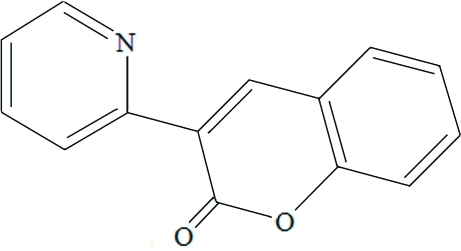


## Experimental

### 

#### Crystal data

C_14_H_9_NO_2_
*M* *_r_* = 223.22
Orthorhombic, 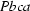

*a* = 7.1107 (3) Å
*b* = 13.9635 (5) Å
*c* = 21.2867 (9) Å
*V* = 2113.56 (15) Å^3^
*Z* = 8
Cu *K*α radiation
μ = 0.77 mm^−1^
*T* = 293 K
0.31 × 0.22 × 0.11 mm

#### Data collection

Siemens SMART CCD diffractometer
Absorption correction: multi-scan (*SADABS*; Sheldrick, 1996 ▶) *T*_min_ = 0.795, *T*_max_ = 0.920
4495 measured reflections
2055 independent reflections
1581 reflections with *I* > 2σ(*I*)
*R*_int_ = 0.021

#### Refinement

*R*[*F* ^2^ > 2σ(*F* ^2^)] = 0.072
*wR*(*F* ^2^) = 0.230
*S* = 1.09
2055 reflections
154 parameters
H-atom parameters constrained
Δρ_max_ = 0.55 e Å^−3^
Δρ_min_ = −0.43 e Å^−3^

### 

Data collection: *SMART* (Siemens, 1996 ▶); cell refinement: *SAINT* (Siemens, 1996 ▶); data reduction: *SAINT*; program(s) used to solve structure: *SHELXS97* (Sheldrick, 2008 ▶); program(s) used to refine structure: *SHELXL97* (Sheldrick, 2008 ▶); molecular graphics: *SHELXTL* (Sheldrick, 2008 ▶); software used to prepare material for publication: *SHELXTL*.

## Supplementary Material

Crystal structure: contains datablocks global, I. DOI: 10.1107/S1600536810039796/hb5667sup1.cif

Structure factors: contains datablocks I. DOI: 10.1107/S1600536810039796/hb5667Isup2.hkl

Additional supplementary materials:  crystallographic information; 3D view; checkCIF report

## Figures and Tables

**Table 1:** Hydrogen-bond geometry (Å, °)

*D*—H⋯*A*	*D*—H	H⋯*A*	*D*⋯*A*	*D*—H⋯*A*
C11—H11⋯O2	0.93	2.25	2.875 (3)	124
C12—H12⋯O2^i^	0.93	2.50	3.318 (3)	147

Symmetry code: (i) 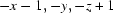.

**Fig. 1.**
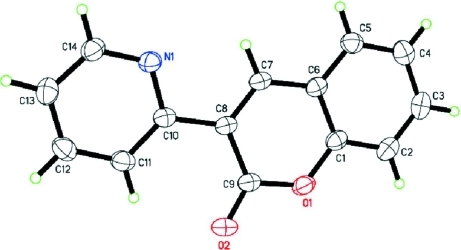
The molecular structure of (I) with displacement ellipsoids for the non-hydrogen atomes drawn at the 30% probability level.

**Fig. 2.**
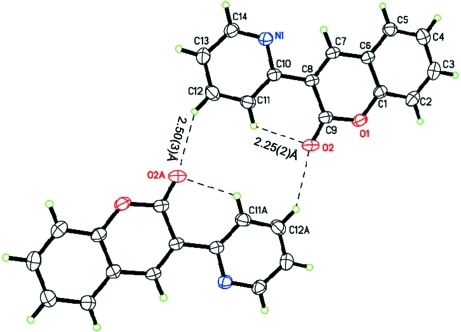
Intramolecular and intermolecular C—H···O hydrogen bonds.

**Fig. 3.**
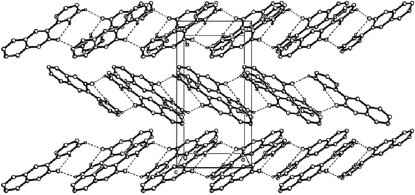
Packing diagram of the title compound along a axis and b axis. H atoms are omitted for clarity.

## supplementary crystallographic information

### Comment

Coumarins are an important class of organic compounds, which have been extensively investigated due to their applications in biological, chemical and physical fields (Walshe, *et al.*, 1997; Fylaktakidou, *et al.*, 2004; Yu, *et al.*, 2010; Trenor, *et al.*, 2004). The photophysical and spectroscopic properties of the coumarin derivatives can be readily modified by the introduction of substituents in parent coumarin, converting themselves into more useful products and more flexibility to fit well in various applications (Griffiths, *et al.*, 1995; Yu, *et al.*, 2006). Among the substituted coumarins, heterocyclic groups at the 3-position have given rise to many derivatives of biological and structural importance. For example, 3-pyridyl substituted coumarins are not only known for their diverse physiological activities (Moffett, *et al.*, 1964), but also have outstanding optical properties including high quantum yields and superior photostability (Yu, *et al.*, 2010). In addition, 3-pyridyl substituted coumarins have attracted considerable interest due to their use as ligands for Ir (III) complexes which possess higher quantum yields and much higher brightnesses (Ren, *et al.*, 2008; Ren, *et al.*, 2010). In this paper, we report the synthesis and crystal structure of 3-(pyridin-2-yl)coumarin.

The molecular structure of the title compound and the *ORTEP* structure is shown in Fig.1. The bond lengths and angles in the molecule are within normal ranges (Allen *et al.*, 1987). Both the pyrone and benzene rings in the coumarin motif are essentially planar. The dihedral angle between them is 1.40 (2)°, thus the coumarin moiety is essentially planar. The pyridine ring makes an angle of 10.40 (3)° with the pyrone ring, they are not coplanar.

The crystal structure is stabilized by intramolecular and intermolecular C—H···O hydrogen bonds (Fig. 2). Specially, the molecules form one-dimensional chains through intermolecular C12—H12···O2 hydrogen bonds with a motif fashion of *R*^2^_2_(14) (Fig. 3).

### Experimental

Salicylaldehyde (0.1 mol) and pyridine-2-acetonitrile (0.1 mol) were dissolved in 30 ml of anhydrous alcohol, and then piperidine (0.1 ml) was added stepwise under ice bath. The mixture was stirred for 12 h at room temperature, then treated with HCl (50 ml, 3.5%) and refluxed for 10 h to hydrolyze the iminocoumarin. When the reaction was finished, the acidic solution was neutralized with aqueous ammonia until the pH was 7. The precipitate was filtered off and recrystallized from methanol to afford the title compound. m.p. 416–417 K. IR (KBr pellet, cm^-1^): 3042 (aryl-CH), 1723 (C=O, lactone), 1605 (C=C), 1579, 1462, 1244, 1109, 1089; ^1^H-NMR (500 MHz, CDCl_3_): 8.87 (s, 1H, H-4), 8.64 (d, 1H, J = 5.4, H-6'), 8.32 (d, 1H, J = 8.2, H-3'), 7.87–7.82 (m, 2H, Aryl-H), 7.63 (t, 1H, J = 8.4, Aryl-H), 7.42–7.34 (m, 3H, Aryl-H).

Colourless blocks of (I) were obtained by slow evaporation of the methanol solution at room temperature.

### Refinement

Non-H atoms were refined anisotropically. H atoms were treated as riding atoms with distances C—H = 0.93 Å (ArH). The isotropic displacement parameters for all H atoms were set equal to 1.2 *U*_eq_ of the carrier atom.

### Crystal data

**Table:**

C_14_H_9_NO_2_	*D*_x_ = 1.403 Mg m^−^^3^
*M**_r_* = 223.22	Melting point = 416–417 K
Orthorhombic, *P**b**c**a*	Cu *K*α radiation, λ = 1.54184 Å
*a* = 7.1107 (3) Å	Cell parameters from 4495 reflections
*b* = 13.9635 (5) Å	θ = 4.2–72.5°
*c* = 21.2867 (9) Å	µ = 0.77 mm^−^^1^
*V* = 2113.56 (15) Å^3^	*T* = 293 K
*Z* = 8	Block, colourless
*F*(000) = 928	0.31 × 0.22 × 0.11 mm

### Data collection

**Table:**

Siemens SMART CCD diffractometer	2055 independent reflections
Radiation source: fine-focus sealed tube	1581 reflections with *I* > 2σ(*I*)
graphite	*R*_int_ = 0.021
φ and ω scans	θ_max_ = 72.5°, θ_min_ = 4.2°
Absorption correction: multi-scan (*SADABS*; Sheldrick, 1996)	*h* = −5→8
*T*_min_ = 0.795, *T*_max_ = 0.920	*k* = −15→17
4495 measured reflections	*l* = −16→26

### Refinement

**Table:**

Refinement on *F*^2^	Primary atom site location: structure-invariant direct methods
Least-squares matrix: full	Secondary atom site location: difference Fourier map
*R*[*F*^2^ > 2σ(*F*^2^)] = 0.072	Hydrogen site location: inferred from neighbouring sites
*wR*(*F*^2^) = 0.230	H-atom parameters constrained
*S* = 1.09	*w* = 1/[σ^2^(*F*_o_^2^) + (0.1725*P*)^2^] where *P* = (*F*_o_^2^ + 2*F*_c_^2^)/3
2055 reflections	(Δ/σ)_max_ < 0.001
154 parameters	Δρ_max_ = 0.55 e Å^−^^3^
0 restraints	Δρ_min_ = −0.43 e Å^−^^3^

### Special details

**Table:**

Geometry. All e.s.d.'s (except the e.s.d. in the dihedral angle between two l.s. planes) are estimated using the full covariance matrix. The cell e.s.d.'s are taken into account individually in the estimation of e.s.d.'s in distances, angles and torsion angles; correlations between e.s.d.'s in cell parameters are only used when they are defined by crystal symmetry. An approximate (isotropic) treatment of cell e.s.d.'s is used for estimating e.s.d.'s involving l.s. planes.
Refinement. Refinement of *F*^2^ against ALL reflections. The weighted *R*-factor *wR* and goodness of fit *S* are based on *F*^2^, conventional *R*-factors *R* are based on *F*, with *F* set to zero for negative *F*^2^. The threshold expression of *F*^2^ > σ(*F*^2^) is used only for calculating *R*-factors(gt) *etc*. and is not relevant to the choice of reflections for refinement. *R*-factors based on *F*^2^ are statistically about twice as large as those based on *F*, and *R*- factors based on ALL data will be even larger.

### Fractional atomic coordinates and isotropic or equivalent isotropic displacement parameters (Å^2^)

**Table:**

	*x*	*y*	*z*	*U*_iso_*/*U*_eq_	
C1	0.2429 (4)	0.14529 (15)	0.41263 (10)	0.0430 (6)	
C2	0.3906 (4)	0.20503 (18)	0.42654 (12)	0.0544 (7)	
H2	0.3935	0.2381	0.4645	0.065*	
C3	0.5343 (4)	0.21543 (19)	0.38377 (14)	0.0596 (7)	
H3	0.6346	0.2559	0.3928	0.072*	
C4	0.5307 (4)	0.1655 (2)	0.32686 (13)	0.0577 (7)	
H4	0.6279	0.1730	0.2980	0.069*	
C5	0.3826 (4)	0.10503 (18)	0.31356 (11)	0.0505 (6)	
H5	0.3811	0.0716	0.2758	0.061*	
C6	0.2348 (3)	0.09346 (15)	0.35618 (10)	0.0408 (5)	
C7	0.0765 (3)	0.03304 (15)	0.34618 (9)	0.0410 (5)	
H7	0.0717	−0.0030	0.3095	0.049*	
C8	−0.0669 (3)	0.02542 (14)	0.38726 (9)	0.0393 (5)	
C9	−0.0573 (4)	0.08126 (16)	0.44606 (10)	0.0448 (6)	
C10	−0.2310 (3)	−0.03764 (15)	0.37410 (9)	0.0396 (5)	
C11	−0.3687 (4)	−0.06205 (18)	0.41772 (11)	0.0497 (6)	
H11	−0.3634	−0.0377	0.4583	0.060*	
C12	−0.5124 (4)	−0.1222 (2)	0.40050 (12)	0.0562 (7)	
H12	−0.6053	−0.1386	0.4293	0.067*	
C13	−0.5179 (4)	−0.15823 (19)	0.34010 (12)	0.0541 (6)	
H13	−0.6128	−0.1997	0.3273	0.065*	
C14	−0.3768 (4)	−0.13020 (18)	0.29945 (11)	0.0507 (6)	
H14	−0.3802	−0.1538	0.2586	0.061*	
N1	−0.2370 (3)	−0.07198 (14)	0.31462 (8)	0.0462 (5)	
O1	0.0984 (3)	0.13797 (12)	0.45491 (8)	0.0503 (5)	
O2	−0.1716 (3)	0.08250 (16)	0.48780 (9)	0.0668 (6)	

### Atomic displacement parameters (Å^2^)

**Table:**

	*U*^11^	*U*^22^	*U*^33^	*U*^12^	*U*^13^	*U*^23^
C1	0.0534 (12)	0.0348 (10)	0.0408 (11)	0.0064 (9)	−0.0047 (9)	−0.0012 (8)
C2	0.0652 (15)	0.0419 (11)	0.0562 (13)	0.0006 (11)	−0.0111 (11)	−0.0058 (9)
C3	0.0567 (14)	0.0453 (12)	0.0768 (16)	−0.0083 (11)	−0.0103 (13)	0.0041 (11)
C4	0.0518 (13)	0.0560 (14)	0.0652 (15)	−0.0017 (12)	0.0045 (12)	0.0062 (11)
C5	0.0541 (13)	0.0494 (12)	0.0479 (12)	0.0035 (10)	0.0039 (10)	−0.0010 (9)
C6	0.0469 (12)	0.0361 (10)	0.0394 (10)	0.0070 (8)	−0.0045 (8)	−0.0012 (8)
C7	0.0508 (12)	0.0398 (10)	0.0324 (10)	0.0056 (9)	−0.0016 (8)	−0.0047 (7)
C8	0.0490 (12)	0.0364 (9)	0.0325 (9)	0.0065 (8)	−0.0011 (8)	−0.0021 (8)
C9	0.0533 (13)	0.0439 (11)	0.0372 (10)	0.0057 (10)	0.0034 (9)	−0.0063 (8)
C10	0.0483 (12)	0.0365 (10)	0.0341 (10)	0.0059 (8)	−0.0010 (8)	0.0021 (7)
C11	0.0586 (14)	0.0499 (12)	0.0406 (11)	0.0023 (11)	0.0081 (10)	−0.0003 (9)
C12	0.0564 (14)	0.0587 (14)	0.0535 (13)	−0.0035 (12)	0.0129 (11)	0.0070 (10)
C13	0.0563 (14)	0.0494 (12)	0.0566 (13)	−0.0093 (11)	−0.0074 (11)	0.0059 (10)
C14	0.0626 (15)	0.0479 (12)	0.0416 (11)	−0.0051 (10)	−0.0051 (10)	−0.0017 (9)
N1	0.0555 (11)	0.0474 (10)	0.0357 (9)	−0.0036 (8)	0.0007 (8)	−0.0011 (7)
O1	0.0630 (11)	0.0465 (9)	0.0415 (8)	−0.0009 (7)	0.0000 (7)	−0.0119 (6)
O2	0.0746 (13)	0.0742 (13)	0.0514 (10)	−0.0078 (11)	0.0216 (9)	−0.0240 (8)

### Geometric parameters (Å, °)

**Table:**

C1—O1	1.369 (3)	C8—C9	1.476 (3)
C1—C2	1.374 (4)	C8—C10	1.489 (3)
C1—C6	1.404 (3)	C9—O2	1.204 (3)
C2—C3	1.376 (4)	C9—O1	1.375 (3)
C2—H2	0.9300	C10—N1	1.355 (3)
C3—C4	1.398 (4)	C10—C11	1.392 (3)
C3—H3	0.9300	C11—C12	1.373 (4)
C4—C5	1.379 (4)	C11—H11	0.9300
C4—H4	0.9300	C12—C13	1.381 (4)
C5—C6	1.398 (3)	C12—H12	0.9300
C5—H5	0.9300	C13—C14	1.382 (4)
C6—C7	1.423 (3)	C13—H13	0.9300
C7—C8	1.347 (3)	C14—N1	1.324 (3)
C7—H7	0.9300	C14—H14	0.9300
			
O1—C1—C2	118.5 (2)	C7—C8—C10	121.18 (18)
O1—C1—C6	119.5 (2)	C9—C8—C10	120.52 (19)
C2—C1—C6	121.9 (2)	O2—C9—O1	115.7 (2)
C1—C2—C3	119.3 (2)	O2—C9—C8	127.0 (2)
C1—C2—H2	120.4	O1—C9—C8	117.2 (2)
C3—C2—H2	120.4	N1—C10—C11	121.0 (2)
C2—C3—C4	120.5 (2)	N1—C10—C8	114.20 (19)
C2—C3—H3	119.8	C11—C10—C8	124.82 (19)
C4—C3—H3	119.8	C12—C11—C10	119.7 (2)
C5—C4—C3	119.8 (3)	C12—C11—H11	120.2
C5—C4—H4	120.1	C10—C11—H11	120.2
C3—C4—H4	120.1	C11—C12—C13	119.5 (2)
C4—C5—C6	120.8 (2)	C11—C12—H12	120.2
C4—C5—H5	119.6	C13—C12—H12	120.2
C6—C5—H5	119.6	C12—C13—C14	117.4 (2)
C5—C6—C1	117.7 (2)	C12—C13—H13	121.3
C5—C6—C7	124.5 (2)	C14—C13—H13	121.3
C1—C6—C7	117.8 (2)	N1—C14—C13	124.5 (2)
C8—C7—C6	123.25 (19)	N1—C14—H14	117.8
C8—C7—H7	118.4	C13—C14—H14	117.8
C6—C7—H7	118.4	C14—N1—C10	118.0 (2)
C7—C8—C9	118.3 (2)	C1—O1—C9	123.88 (17)
			
O1—C1—C2—C3	−178.1 (2)	C10—C8—C9—O1	−179.93 (19)
C6—C1—C2—C3	0.7 (4)	C7—C8—C10—N1	−10.5 (3)
C1—C2—C3—C4	−0.3 (4)	C9—C8—C10—N1	169.58 (19)
C2—C3—C4—C5	−0.3 (4)	C7—C8—C10—C11	168.8 (2)
C3—C4—C5—C6	0.5 (4)	C9—C8—C10—C11	−11.1 (3)
C4—C5—C6—C1	−0.1 (3)	N1—C10—C11—C12	0.4 (4)
C4—C5—C6—C7	179.7 (2)	C8—C10—C11—C12	−178.9 (2)
O1—C1—C6—C5	178.3 (2)	C10—C11—C12—C13	0.3 (4)
C2—C1—C6—C5	−0.5 (3)	C11—C12—C13—C14	−0.7 (4)
O1—C1—C6—C7	−1.6 (3)	C12—C13—C14—N1	0.4 (4)
C2—C1—C6—C7	179.7 (2)	C13—C14—N1—C10	0.2 (4)
C5—C6—C7—C8	−177.9 (2)	C11—C10—N1—C14	−0.7 (3)
C1—C6—C7—C8	1.8 (3)	C8—C10—N1—C14	178.7 (2)
C6—C7—C8—C9	−1.1 (3)	C2—C1—O1—C9	179.5 (2)
C6—C7—C8—C10	178.96 (18)	C6—C1—O1—C9	0.7 (3)
C7—C8—C9—O2	−179.5 (3)	O2—C9—O1—C1	179.8 (2)
C10—C8—C9—O2	0.4 (4)	C8—C9—O1—C1	0.1 (3)
C7—C8—C9—O1	0.2 (3)		

### Hydrogen-bond geometry (Å, °)

**Table:**

*D*—H···*A*	*D*—H	H···*A*	*D*···*A*	*D*—H···*A*
C11—H11···O2	0.93	2.25	2.875 (3)	124
C12—H12···O2^i^	0.93	2.50	3.318 (3)	147

Symmetry codes: (i) −*x*−1, −*y*, −*z*+1.
